# Vasodilation Elicited by Isoxsuprine, Identified by High-Throughput Virtual Screening of Compound Libraries, Involves Activation of the NO/cGMP and H_2_S/K_ATP_ Pathways and Blockade of α_1_-Adrenoceptors and Calcium Channels

**DOI:** 10.3390/molecules24050987

**Published:** 2019-03-11

**Authors:** Daniella Medina-Ruiz, Berenice Erreguin-Luna, Francisco J. Luna-Vázquez, Antonio Romo-Mancillas, Alejandra Rojas-Molina, César Ibarra-Alvarado

**Affiliations:** 1 Posgrado en Ciencias Químico Biológicas, Facultad de Química, Universidad Autónoma de Querétaro, Cerro de las Campanas S/N, Querétaro C.P. 76010, Mexico; daniellamedinar@gmail.com; 2 Laboratorio de Investigación Química y Farmacológica de Productos Naturales, Facultad de Química, Universidad Autónoma de Querétaro, Centro Universitario, Querétaro 76010, Mexico; bereel@outlook.com (B.E.-L.); fjlunavz@yahoo.com.mx (F.J.L.-V.); rojasa@uaq.mx (A.R.-M.); 3 Laboratorio de Diseño Asistido por Computadora y Síntesis de Fármacos, Facultad de Química, Universidad Autónoma de Querétaro, Centro Universitario, Querétaro 76010, Mexico; ruben.romo@uaq.mx

**Keywords:** high-throughput virtual screening, isoxsuprine, NO/cGMP and H_2_S/K_ATP_ pathways, calcium channels, vasodilation

## Abstract

Recently, our research group demonstrated that uvaol and ursolic acid increase NO and H_2_S production in aortic tissue. Molecular docking studies showed that both compounds bind with high affinity to endothelial NO synthase (eNOS) and cystathionine gamma-lyase (CSE). The aim of this study was to identify hits with high binding affinity for the triterpene binding-allosteric sites of eNOS and CSE and to evaluate their vasodilator effect. Additionally, the mechanism of action of the most potent compound was explored. A high-throughput virtual screening (HTVS) of 107,373 compounds, obtained from four ZINC database libraries, was performed employing the crystallographic structures of eNOS and CSE. Among the nine top-scoring ligands, isoxsuprine showed the most potent vasodilator effect. Pharmacological evaluation, employing the rat aorta model, indicated that the vasodilation produced by this compound involved activation of the NO/cGMP and H_2_S/K_ATP_ signaling pathways and blockade of α_1_-adrenoceptors and L-type voltage-dependent Ca^2+^ channels. Incubation of aorta homogenates in the presence of isoxsuprine caused 2-fold greater levels of H_2_S, which supported our preliminary in silico data. This study provides evidence to propose that the vasodilator effect of isoxsuprine involves various mechanisms, which highlights its potential to treat a wide variety of cardiovascular diseases.

## 1. Introduction

High blood pressure plays a major role in the occurrence of cardiovascular diseases (CVD), which represent the main cause of death in the world [1,2]. Usually, hypertension treatment requires the employment of multiple antihypertensive drugs, many of which have low therapeutic effectiveness and cause severe adverse effects [1,3,4,5].

Hypertension has been associated with endothelial dysfunction, which is characterized by a disruption in the synthesis and/or release of endothelium-derived relaxing factors [6,7,8,9,10], such as the gasotransmitters NO and H_2_S, whose participation in regulating vasodilation is critical [11,12,13,14,15]. In recent years, the interest in finding novel drugs that either donate NO and H_2_S or induce their synthesis, or both, has increased [14,16,17]. 

Earlier in silico studies suggested that naturally occurring triterpenes, such as uvaol and ursolic, morolic, and betulinic acids, display high affinity for binding pockets found in the substrate access to the catalytic site of eNOS and might directly activate it [18,19]. We also previously found, using in silico analysis, that ursolic acid and uvaol bind with high affinity to a site that could act as an allosteric site on CSE [18]. These findings supported the hypothesis that the proposed triterpene-binding sites to both enzymes might represent promissory pharmacological targets in the search of new hit compounds for the development of drugs useful to treat CVD. In this context and considering that high throughput virtual screening (HTVS) of lead-like libraries represents a valuable hit finding strategy for pharmaceutical research and development [20,21,22,23], the aim of the present study was to identify new hits that bind with high affinity to the triterpene-binding putative allosteric sites on eNOS and CSE, through HTVS of commercial natural and synthetic compound libraries. Compounds that showed the highest affinity for the proposed pharmacological targets were evaluated *ex vivo* and participation of the NO/cGMP and H_2_S/K_ATP_ pathways in their vasodilator effect was determined. Once the compound with the most potent vasodilator effect was identified, its mechanism of action was investigated in more detail.

## 2. Results

### 2.1. Virtual Screening

**Figure 1:**
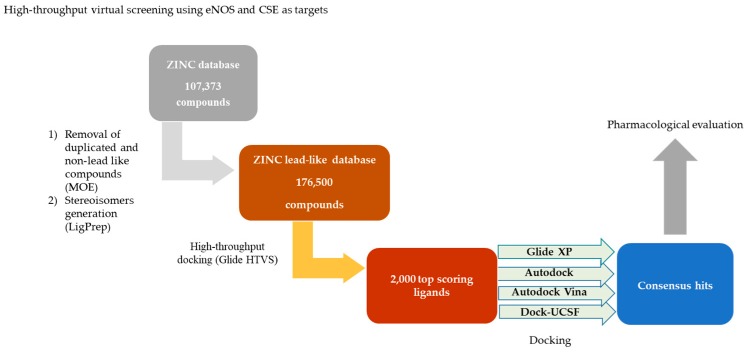
Virtual screening strategy diagram. Once duplicated compounds were removed from the ZINC database, lead-like compounds were selected and high-throughput virtual screening was done. Compounds with the highest scores were docked using Glide XP, Autodock, Autodock Vina, and Dock-UCSF. The consensus hits were identified and subsequently evaluated.

Figure 1 shows the sequence of the screening process. 107,373 compounds, with unique Smiles codes, were obtained from the ZINC database. The MOE software [24] was used in order to exclusively select non-reactive compounds with suitable physico-chemical properties (MW under 500 and less than 5 hydrogen bond donors and 10 hydrogen bond acceptors). The LigPrep program (Schrödinger Release 2015-4) was employed to generate 3D structures of the selected compounds, considering their stereochemistry, protonation states, and tautomeric forms. 

This procedure led to a set of 176,500 lead like structures that were subjected to a docking into the triterpene allosteric binding sites on eNOS and CSE [18], using the GLIDE High-Throughput Virtual Screening (HTVS) docking module (Glide, version 6.2, Schrödinger) [25,26,27]. Virtual screening was performed with the highest-resolution protein structures available from the Protein Data Bank archive, eNOS (PDB: 3NOS) [28] and CSE (PDB: 3COG) [29]. The 2000 top scoring ligands for each of the targets were subsequently docked within the binding site of interest, employing Glide XP [27], AutoDock [30], AutoDock Vina [31], and UCSF-Dock [32]. The nine top ranking compounds with best scores (“consensus hits”; Supplementary materials Table S1) were selected via consensus in all four programs [33].

### 2.2. Determination of the Vasodilator Effect of the Consensus Hits and Involvement of the NO/cGMP and the H_2_S/K_ATP_ Pathways in Their Mechamism of Action

All selected consensus hits induced a significant concentration-dependent relaxation of the rat aorta and reached a 100% of maximum effect. The most potent compounds were isoxsuprine (EC_50_ = 0.046 ± 0.004 μM) and carvedilol (EC_50_ = 0.069 ± 0.003 μM), which turned out to be approximately five-fold less potent that sodium nitroprusside (SNP: EC_50_ = 0.0099 ± 0.001 μM), used as a positive control. 

**Figure 2:**
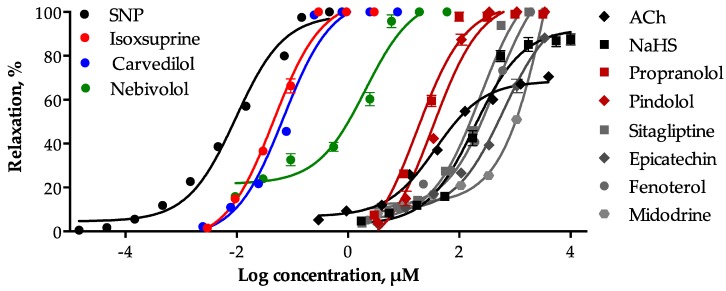
Concentration-response curves of the vasodilator effect of the consensus hits and the positive controls (SNP, ACh, and NaHS). Values are expressed as mean ± SEM (*n* = 6).

Nebivolol showed an EC_50_ = 2.014 ± 0.215 μM, whereas, sitagliptin, fenoterol, midodrin, epicatechin, pindolol, and propranolol showed EC_50_ values higher than 18 µM. The concentration-response curves (CRC) of the vasodilator effect elicited by the consensus hits and the positive controls [SNP, acetylcholine (ACh), and sodium hydrosulfide (NaHS)] are shown in Figure 2 and their EC_50_ and Emax values are summarized in Table 1.

Inhibition of eNOS with 100 µM *N*^G^-nitro-L-arginine methyl ester (L-NAME) significantly shifted to the right the CRC of the consensus hits, with the consequent increase in their respective EC_50_ values (Table 2). Although it had been previously described that NO is involved in the vasorelaxant effect of carvedilol [34], nebivolol [35], propranolol [36], pindolol [37], and epicatechin [38], this is the first report, which shows that activation of the NO/cGMP pathway contributes to the vasodilator effect elicited by midodrine, sitagliptin, fenoterol, and isoxsuprine. On the other hand, the vasodilator effect of all the consensus hits, with the exception of nebivolol and propranolol, was reduced by 10 mM DL-propargylglycine (PAG), which was evidenced by the increase in their EC_50_ values (Table 2). These results suggested that the vasodilation elicited by the consensus hits involves activation of eNOS and/or CSE. 

**Figure 3:**
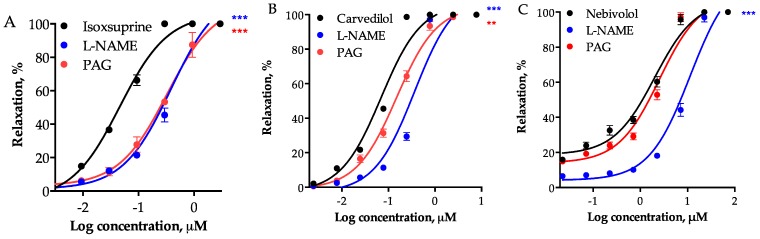
Vasodilatory effect of (**A**) isoxsuprine, (**B**) carvedilol, and (**C**) nebivolol in the absence (control) and presence of inhibitors of eNOS and CSE (L-NAME, 100 µM and PAG, 10 mM, respectively). Data are means ± SEM (*n* = 6). Statistical analysis was made using a one-way ANOVA, followed by Dunnett’s post hoc test (** *p* < 0.001, *** *p* < 0.0001).

**Figure 4:**
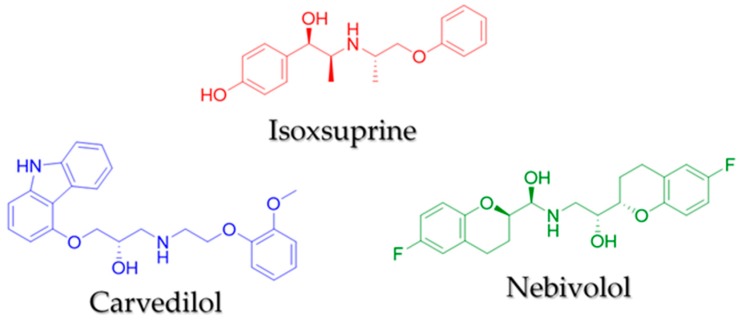
Chemical structures of the most potent compounds: isoxsuprine, carvedilol, and nebivolol.

Figure 3 shows participation of the NO/cGMP and H_2_S/K_ATP_ pathways in the vasodilator effect elicited by isoxsuprine, carvedilol, and nebivolol, the three most potent consensus hits (Figure 4). Both biochemical pathways importantly contribute to isoxsuprine- and carvedilol- induced vasodilation. However, the vasodilator effect elicited by nebivolol is independent of the H_2_S/K_ATP_ pathway. 

### 2.3. Virtual Pharmacophoric Elements Identification and General Protein-Ligand Interaction Model

**Figure 5:**
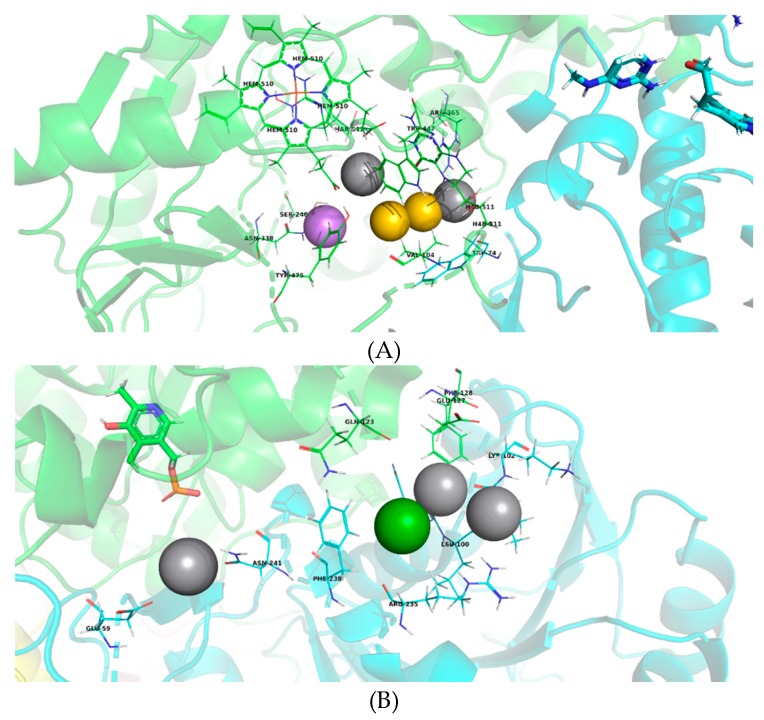
Model of pharmacophoric elements with (**A**) eNOS (3NOS structure) and (**B**) CSE (3COG structure). Hydrogen bond donors are represented in gray; aromatic-type interactions in green; hydrophobic interactions in yellow and an interaction as bridge acceptor of hydrogen in purple.

Considering that individual identification of the interactions relevant for the binding of the consensus hits to eNOS and CSE would be limited to that particular compound and thus would render limited information about the structural requirements for best binding, we conducted a search of pharmacophoric elements in the best protein-ligand complexes obtained by consensus docking. Thereafter, we applied Partitioning Around Medoids (PAM) to cluster these elements in order to identify the main residues and protein-ligand interactions essential for increased affinity for both enzymes (Figure 5). This statistical analysis, which is not a formal pharmacophore model, will be useful for future identification of active compounds.

In the case of eNOS, the main amino acid residues that interact with the consensus hits are located both on the substrate access channel and within the catalytic site. The tested molecules showed two interactions between hydrogen bond donors and heme (HEM) and tetrahydrobiopterin (H4B) cofactors (represented in gray), as well as hydrophobic interactions with Trp447 and Val104 (in yellow), and an additional interaction between a hydrogen bond acceptor and Asn338 (in purple). These results suggest that the consensus hits bind to eNOS at different amino acid residues to which uvaol and ursolic acid bind, most likely due to their structural differences, since unlike triterpenoids that are mainly hydrophobic, the consensus hits have more hydrogen bond donors and acceptors.

On the other hand, the consensus hits were found to interact with amino acids that belong to subunits A and B of CSE, which supports our proposal that the ligand binding site is located at the interface between these two subunits. These residues are positioned within a 12.5 Å radius of the pyridoxal phosphate of chain A (Figure 5B), which could be considered a large distance to influence the catalytic site. However, the binding site is placed very close to the substrate access channel to the catalytic site, which is formed within the A–B interface of this homotetrametic enzyme. Hydrogen bonding interactions are observed between hydrogen bond donors located in the ligands and Glu59, His99, and Leu101 of CSE (in gray). An additional aromatic interaction with Phe238 (in green) was found. These findings suggest that the consensus hits bind to the proposed binding site of triterpenes found in our previous works on CSE and could play a role as allosteric activators of this enzyme.

### 2.4. Molecular Dynamics (MD) of Isoxuprine-CSE Interaction

**Figure 6:**
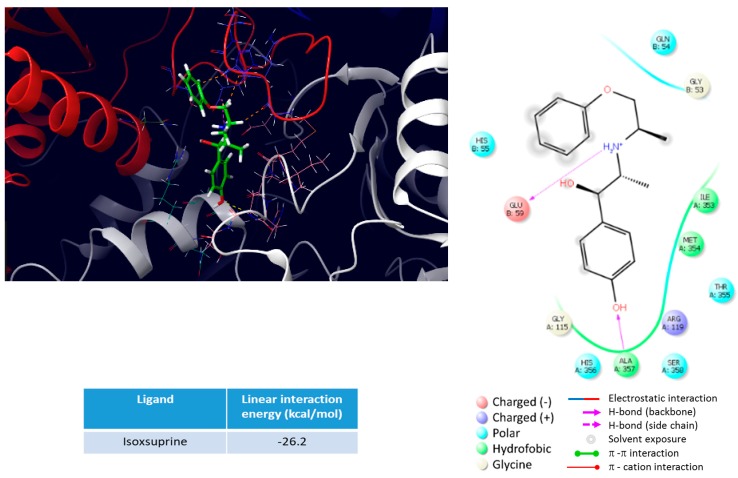
Isoxsuprine-CSE interactions during MD simulations using GROMACS.

Considering the vasodilator potency of isoxsuprine and the results obtained from molecular docking, which suggested that this compound displays high binding affinity for CSE, we conducted a MD simulation of isoxsuprine-CSE interaction (the most potent vasodilator) and fenoterol-CSE interaction (this compound belongs to the group of less potent vasodilators, Table 2). The plot of calculated binding energy (CBE) (Figure S1, Supplementary materials) indicated that the isoxuprine-CSE complex remains in equilibrium for approximately 5 ns, and subsequently this compound gradually loses its affinity for the enzyme. On the other hand, fenoterol showed a favorable affinity for CSE at the start of the simulation, to subsequently lose it faster than isoxsuprine does. In the stability period (1–5 ns), the fenoterol-CSE complex has a mean LIE of 1.83 kcal/mol, which is lower than the mean LIE of −26.2 kcal/mol calculated for isoxsuprine-CSE in the same period (Figure 6).

Our ex vivo assays clearly demonstrated that the H_2_S/K_ATP_ pathway is involved in isoxsuprine-induced vasodilation, indicating that although in silico MD simulation showed that isoxsuprine affinity for CSE decreases during the simulation period, ligand-enzyme interactions are significant enough to increase enzymatic activity, which eventually contributes to produce an important vasodilation. Contrastingly, fenoterol, which according to the MD simulation quickly loses affinity for CSE, displayed a significantly lower vasodilation.

Figure 6 shows isoxsuprine-CSE interactions during MD simulations using GROMACS. Isoxsuprine-CSE complex lost some of the binding interactions previously visualized through molecular docking. However, MD simulation data confirmed that isoxsuprine establishes interactions with A and B chain residues by forming hydrogen bonds between its amino group and a glutamic acid residue (Glu59) and its phenolic hydroxyl group and an alanine residue (Ala357). These bindings are located at the interface of A and B subunits of CSE at the previously identified as a triterpene-binding site [18]. Noteworthy, this site was preliminarily tested by molecular dynamics simulations carried out with the naturally occurring triterpenes uvaol and oleanolic acid [39], which elicited a medium and almost nil H_2_S-dependent vasodilatory activity, respectively. According to those simulations, uvaol and oleanolic acid form a complex with CSE with a mean LIE of −0.73 and 33.88 kcal/mol, respectively. These results, together with the ones we obtained in the present study for fenoterol and isoxsuprine, suggests a possible correlation between the vasodilator effect and the theoretical LIE value, which predicts the binding affinity of docked compounds with CSE. 

### 2.5. Increase in H_2_S Levels Elicited by Isoxsuprine

**Figure 7:**
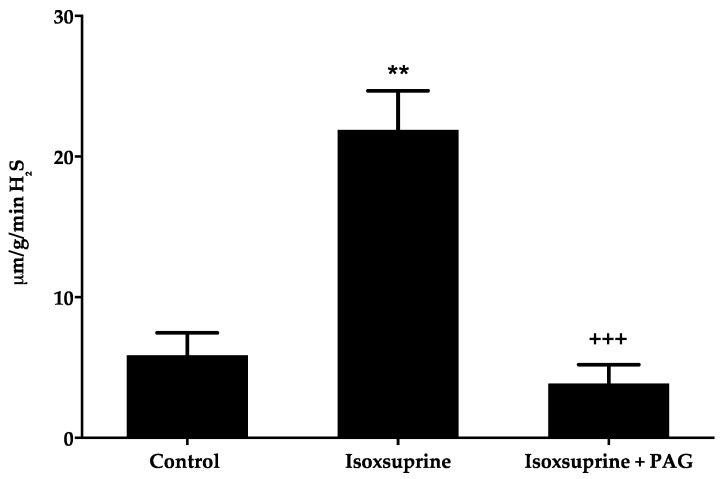
Total H_2_S production (measured as sulfides) induced by isoxsuprine in rat aortic homogenates in the absence or presence of PAG. Statistical analysis was made using a one-way ANOVA, followed by a Tukey’s test (** *p* = 0.0001 isoxsuprine vs. control; ^+++^ *p* < 0.0001 isoxsuprine vs. isoxsuprine + PAG).

In order to confirm whether direct stimulation of CSE participated, at least partly, in isoxsuprine-induced vasodilation, as suggested by our in silico study, H_2_S levels in aorta homogenates were measured. We found that incubation of rat aorta rings with isoxsuprine resulted in approximately two-fold increased levels of this gasotransmitter (Figure 7). As expected, when aortic rings were simultaneously incubated with isoxsuprine and PAG, a drastic reduction in H_2_S levels was observed. These results supported the hypothesis that isoxsuprine might directly activate CSE.

### 2.6. Participation of Other Endothelial-Derived Relaxing Factors in the Vasodilator Effect of Isoxsuprine

**Figure 8:**
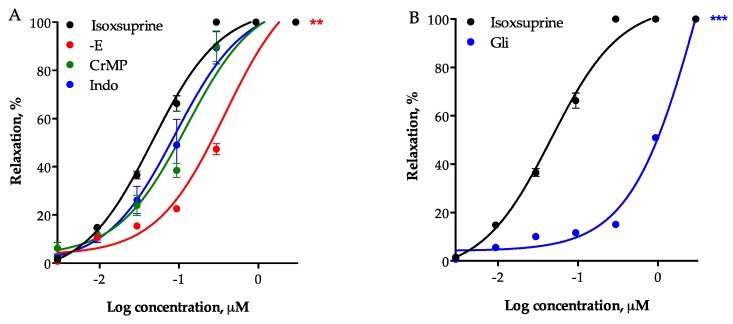
(**A**) Vasodilatory effect of isoxsuprine in the presence (+E) or absence (-E) of endothelium or in the presence of the heme oxygenase inhibitor, chromium mesoporphyrin IX (CrMP) (15 μM) or the cyclooxygenase inhibitor, indomethacin (Indo) (10 μM). Statistical analysis was made using a one-way ANOVA, followed by Dunnett’s post hoc test (** *p* < 0.001 vs. control). (**B**) Vasodilatory effect of isoxsuprine in the absence (control) or presence of the ATP-dependent potassium channel (K_ATP_) inhibitor, glibenclamide (Gli) (1 μM). Statistical analysis was made using t-test with Welch´s correction (*** *p* < 0.0001).

Denudation of aortic rings significantly reduced the vasodilator effect of isoxsuprine (EC_50_ = 0.3781 ± 0.019 μM), further supporting involvement of the NO/cGMP and H_2_S/K_ATP_ pathways in its vasodilator effect. However, considering that eNOS and CSE inhibition did not completely abolish isoxsuprine-induced vasodilation, the role of other endothelium-derived vasodilators, such as CO and prostacyclin (PGI_2_) was investigated. Neither inhibition of heme oxygenase (HO) with chromium mesoporphyrin IX, nor inhibition of cyclooxygenase (COX) with indomethacin significantly reduced vasodilatory effect of isoxsuprine (Figure 8A). In contrast, the vasorelaxant effect of isoxsuprine was significantly reduced by blockade of ATP-dependent potassium channels (K_ATP_) (EC_50_ = 1.152 ± 0.0335 μg/mL; *p* < 0.0001) (Figure 8B), indicating that these channels are involved in this effect.

### 2.7. Participation of β_2_-Adrenoceptor Activation and α_1_-Adrenoceptor Blockade in Isoxsuprine-Induced Vasodilation

**Figure 9:**
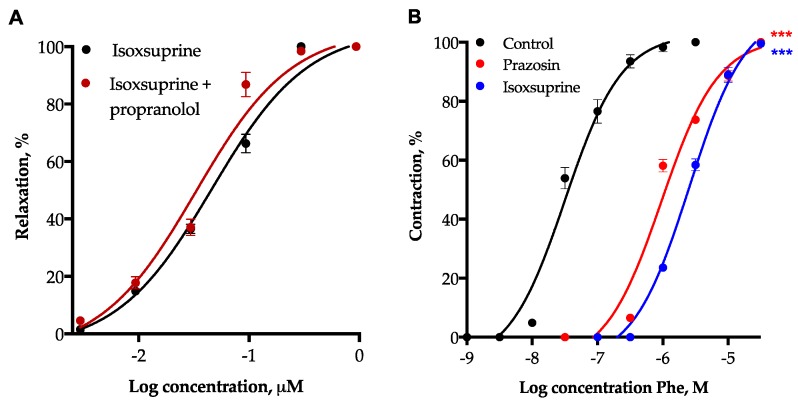
(**A**) Vasodilatory effect of isoxsuprine in the absence and presence of propranol (1 µM), a β-receptor antagonist. (**B**) Vasoconstrictor effect of Phenylephrine (Phe) in the absence (control) or presence of isoxsuprine (1 µM) or prazosin (0.12 µM), used as a positive control. Data are means ± SEM (*n* = 6). Statistical analysis was made using a one-way ANOVA, followed by Dunnett’s post hoc test. Values of *** *p* < 0.0001 were considered as significant.

In order to determine participation of β_2_-adrenoceptors in isoxsuprine-induced vasodilation, aortic rings were incubated with 1 µM propranolol (a non-selective antagonist of β_1_ and β_2_ receptors). Our results indicated that the vasodilator effect produced by isoxsuprine (EC_50_ = 0.046 ± 0.004 µM) doesn´t involve activation of β_2_-adrenergic receptors, as demonstrated by the fact that the mean effective concentration of the vasodilation provoked by isoxsuprine was not significantly modified (*p* = 0.9615) in the presence of 1 µM propranolol (EC_50_ = 0.033 ± 0.003 µM). On the other hand, 1 µM isoxsuprine significantly (*p* < 0.0001) shifted to the right (EC_50_ = 2.56 ± 0.19 µM) the concentration-response curve for the vasoconstrictor effect of phenylephrine (EC_50_ = 0.032 ± 0.003 µM), a specific α_1_-adrenoceptor agonist. A similar behavior was observed when the vasoconstriction provoked by phenylephrine was evaluated in the presence of prazosin (EC_50_ = 0.9 ± 0.057 µM, *p* < 0.0001), a specific inhibitor of α_1_-adrenoceptors (Figure 9B). In summary, the results derived from these pharmacological experiments showed evidence suggesting that blockade of α_1_-adrenoceptors contributes to isoxsuprine-induced vasodilation, while activation of β_2_-adrenoceptors does not participate in the vasodilatory mechanism of this compound. 

### 2.8. Involvement of L-Type Voltage-Dependent Calcium Channels in the Vasodilator Effect of Isoxsuprine

**Figure 10:**
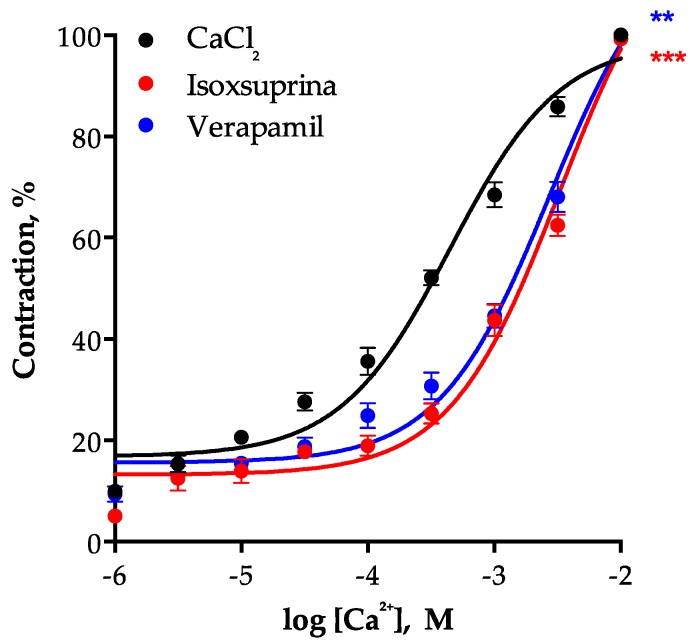
Vasoconstrictor effect of CaCl_2_ in the absence or presence of isoxsuprine or verapamil (positive control). Statistical analysis was made using a one-way ANOVA, followed by Dunnett’s post hoc test (** *p* < 0.001 and *** *p* < 0.0001 vs. control (CaCl_2_)).

To assess participation of L-type voltage-dependent calcium channels (LVCCs) in the vasodilator effect of isoxsuprine, a CRC of the vasoconstrictor effect of CaCl_2_ on isolated rat aorta in the presence of isoxsuprine was constructed (Figure 10). The results showed that this compound is capable of blocking LVCCs, since it shifted the CRC of CaCl_2_-induced aortic contractions (EC_50_ = 0.0004459 ± 0.00003 M) to the right and significantly increased the EC_50_ (0.003256 ± 0.0002 M, *p* < 0.0001). Verapamil, used as a positive control, behaved in a similar manner (EC_50_ = 0.002712 ± 0.0003 M, *p* = 0.0008). 

## 3. Discussion

Our previous studies carried out on ursolic acid and uvaol showed that the vasodilator effect produced by these two natural triterpenes, is mediated by the NO/cGMP and H_2_S/K_ATP_ pathways, possibly by binding to putative allosteric control sites located in NOS and CSE, which provokes direct activation of these enzymes [18]. We therefore proposed that the triterpene binding sites on both enzymes might represent allosteric control sites that could be considered valuable pharmacological targets in the search of new hits for the development of drugs to treat CVD. These hits, very likely structurally different from competitive activators, could act as positive allosteric modulators capable of activating these two important enzymes, without competing with orthosteric ligands [40,41]. In a first approach for detecting new allosteric modulators of eNOS and CSE, we performed a HTVS of commercially-available lead-like compounds, which led to the identification of nine consensus hits that displayed high binding affinity scores for both enzymes. The top scoring hits turned out to be two beta adrenergic agonists (fenoterol and isoxsuprine) [42,43], four beta adrenergic antagonists (carvedilol, nebivolol, propranolol, and pindolol) [37,44,45], an alpha-1 adrenergic agonist (midodrine) [46], a dipeptidyl-peptidase IV inhibitor (sitagliptin) [47], and an antioxidant (epicatechin) [48]. As expected, all nine consensus hits displayed a vasodilator effect on the isolated rat aorta. This is the first report, which shows that activation of the NO/cGMP pathway contributes to the vasodilator effect elicited by midodrine, fenoterol, sitagliptin, and isoxsuprine. The results obtained in the present study, support previous findings, which indicated that the pharmacological effects of fenoterol on tissues other than blood vessels are associated with a rise in NO levels [49,50]. Our results also agree with earlier studies, which demonstrated that sitagliptin, orally administered to diabetic rats, significantly increased NO levels in rat aortas and blood serum [47,51]. The HTVS performed at the triterpene-binding putative allosteric site on eNOS led to the detection of carvedilol, nebivolol, propranolol, pindolol, and epicatechin, whose vasodilatory effect mediated by activation of the NO/cGMP pathway has previously been demonstrated [34,35,36,37,38]. 

It is worth noting that the vasodilator effect of the consensus hits, excepting nebivolol and propranolol, involved activation of the H_2_S/K_ATP_ pathway. Our findings provide a heretofore unknown evidence that the H_2_S/K_ATP_ pathway participates in the vasodilation induced by midodrine, fenoterol, sitagliptin, isoxsuprine, carvedilol, pindolol, and epicatechin. 

Isoxsuprine, carvedilol, and nebivolol displayed the most potent vasodilator effect. The potency of isoxsuprine was similar to that of carvedilol, while that of nebivolol turned out to be more than 30-fold lower. This might be attributed to the fact that the H_2_S/K_ATP_ pathway does not participate in nebivolol-induced vasodilation. Numerous evidences support that H_2_S enhances vascular NO signaling, thus favoring vasodilation [52]. Therefore, it is possible to hypothesize that in the case of isoxsuprine and carvedilol, simultaneous activation of the NO/cGMP and H_2_S/K_ATP_ pathways, which act in a cooperative way [53], leads to an increased vasodilatory effect. 

Nebivolol is widely clinically used, either alone or as an add-on therapy, to treat systemic hypertension or chronic heart failure [54]. This chiral compound is provided as a racemic mixture of two enantiomers. The D isomer is a highly selective β_1_-blocking agent, while the L enantiomer is capable of inducing vascular relaxation enhancing production of NO [55]. The exact mechanism by which L-nevibolol activates endothelial NO synthase has not been elucidated. Some studies provide evidence that this compound increases NO release via endothelial β_3_-receptors [56]. Other authors reported that activation of the NO/cGMP pathway by nebivolol involved activation of β_2-_adrenoceptors [55,57]. Our ex vivo experiments in the presence of L-NAME further confirmed the participation of the NO/cGMP pathway in the vasodilator effect of nebivolol. 

On the other hand, the results of the in silico analysis also showed that carvedilol binds with high affinity to both eNOS and CSE. Consistent with this finding, inhibition of both enzymes significantly decreased the vasodilator effect of this compound. Our results are in agreement with those of previous studies, which indicated that this compound acts on vascular endothelium, provoking NO release [58,59] and enhancing NO bioavailability [45]. Carvedilol, a third-generation and nonselective β-adrenoceptor antagonist, is a licensed drug used for treating patients suffering from heart failure, hypertension, and myocardial ischaemia [60,61]. This compound interacts with multiple biological targets, antagonizing β_1_-adrenergic and NMDA receptors, as well as inhibiting calcium channels [60,62]. However, to date, no study has been reported indicating that the H_2_S/K_ATP_ pathway participates in the antihypertensive effect of this compound. It had already been shown that the vasodilation produced by carvedilol was inhibited, but not completely blocked by L-NAME [63]. Possibly this L-NAME-insensitive vasorelaxation might be attributed to activation of the H_2_S/K_ATP_ pathway, as suggested by our results. Moreover, our in silico analysis showed that stimulation of this pathway could be due to a direct activation of CSE.

On the other hand, of all the consensus hits, isoxsuprine turned out to be the most potent vasodilator, whose mechanism of action involved, to a similar degree, both the NO/cGMP and the H_2_S/K_ATP_ pathways (Figure 4). Unlike, nevibolol and carvedilol, which are successfully used for treating various cardiovascular diseases [54,60,61], isoxsuprine has a restricted therapeutic use and has not been studied systematically. Therefore, we decided to further investigate the mechanism by which this compound exerts its vasorelaxant effect. Our in silico study suggested that this compound is able to bind with high affinity to the triterpene-binding putative allosteric site on CSE. This result was further confirmed in the pharmacological evaluation, which demonstrated that the vasodilatory effect of isoxsuprine was significantly blocked by propargylglycine. Moreover, when measuring H_2_S levels in rat aorta homogenates, after incubation with isoxsuprine, a two-fold increase in the production of this gasotransmitter was observed. All these data supports our proposal that isoxsuprine-induced vasodilation involves activation of the H_2_S/K_ATP_ pathway, very likely through direct interaction with CSE.

Considering that H_2_S directly stimulates ATP-dependent potassium channels (K_ATP_) [64,65], we explored whether these channels were involved in the mechanism of action of isoxsuprine. As expected, glibenclamide significantly decreased the vasodilator effect of this molecule, further supporting that the H_2_S/K_ATP_ pathway underlies its effect. It is known that isoxsuprine activates β_2_-adrenoceptors and blocks α_1_-adrenoceptors, triggering relaxation of smooth muscle [66,67]. Due to this effect isoxsuprine has been employed to treat some pathological conditions, including cerebrovascular insufficiency, Raynaud’s phenomenon, and suppression of premature labor [68]. Interestingly, our experiments, employing the rat aorta model, showed that isoxsuprine-induced vasodilation involves blockade of α_1_-adrenoceptors, but not activation of β_2_-adrenoceptors. Similar results were obtained by Belloli et al. in horse digital artery [67]. In order to explain their findings, those authors hypothesized that smooth muscle from arteries contains low levels of β_2_-adrenoceptors and that isoxsuprine acts as a partial agonist, activating this kind of receptors, only in those tissues where there is a large number of β_2_-adrenoceptors, such as in the fowl caecum [67] and in uterus [69]. Morever, the lack of effect of propranolol pretreatment on the vasodilation elicited by isoxsuprine is consistent with what was found in other vascular and non-vascular tissues including rat jugular vein [70], gravid isolated human myometrium [71], and rat vas deferens [72]. However, what we found in the rat aorta, differ from what Elliot and Soydan (1995) found in isolated equine digital veins, where propranolol slightly inhibited isoxsuprine’s vasodilatory action [73]. In this scenario, it is plausible to propose that propranolol, which is a non-selective β-adrenoceptor antagonist, is not able to significantly block β_2_-adrenoceptor activity in rat aorta. Considering that some studies have provided evidence that β_1_-, β_2_-, and β_3_-adrenoceptors are functionally expressed in vascular endothelial cells and are coupled to activation of the NO/cGMP pathway [37,45], it would be necessary to use specific inhibitors of β_2_-adrenoceptors, such as ICI-118551, in order to determine with greater certainty the role that these type of receptors play in the vasodilatory effect of isoxsuprine. 

Kozlovski et al. showed that coronary vasodilation elicited by both nebivolol and carvedilol does not involve direct activation of beta-2 adrenoceptors, however, they suggested that their metabolites do activate this type of receptors [59]. Considering that it has been suggested that activation of beta-2 adrenoceptors stimulates the NO/cGMP pathway [55,57], it is feasible to hypothesize that the metabolites of these compounds and isoxsuprine interact with these receptors, consequently activating both, the NO/cGMP and/or the H_2_S/K_ATP_ pathways. Evidently, this proposal remains to be confirmed. 

We conducted additional experiments to assess if other signaling pathways were involved in isoxsuprine’s mechanism of action. Inhibition of cyclooxygenase did not significantly reduce isoxsuprine-induced vasodilation. This result differs from those previously found for other beta adrenergic agonists. Inhibition of this enzyme reduced coronary artery dilation caused by terbutaline in hamsters [74], while it produced an increased brachial artery relaxation induced by isoproterenol in humans [75]. These differences might be attributed to both a differential distribution of adrenoceptor subtypes in the various types of arteries, and the use of different experimental species [76]. Inhibition of hemooxygenase also did not decrease the vasodilator effect elicited by isoxsuprine. As far as we have knowledge, there are no reports that indicate whether isoxsuprine-induced vasodilation involves participation of the CO/cGMP pathway. Taken together, these results showed that the PGI_2_/cAMP and CO/cGMP pathways do not contribute to the vasodilator effect produced by this compound. 

Some studies have compared the tocolytic effect of isoxsuprine and that produced by L-type voltage-dependent calcium channel (LVCC) blockers, such as nifedipine [77,78]. However, at present there are not reports about the effect of isoxsuprine on LVCC in rat aorta. We found that, similarly to what happened with verapamil, isoxsuprine shifted to the right the concentration-response curve of the vasoconstrictor effect of calcium chloride. This finding evidenced that the vasodilation evoked by isoxsuprine also involves the blocking of LVCCs. This effect was hitherto unknown. The blockade of LVCCs reduces the flow of extracellular calcium to the vascular smooth muscle cells, increasing vasodilation, which finally leads to a decrease in blood pressure [79]. Diminution of blood pressure, resulting from LVCC blockade by nifedipine or verapamil, is more pronounced in patients with hypertension than in individuals with normal blood pressure, which indicates that LVCC blockers may be considered as specific antihypertensive agents [80]. Moreover, it is a well-demonstrated fact that the combined actions of basal NO release and calcium antagonism results in an inhibition of vasoconstriction, which is greater than the additive effects of both events [81]. A co-cristallized structure of LVCC-verapamil complex provided evidence suggesting that verapamil binds within the pore of the channel [82]. Considering the structural similarity between isoxsuprine and verapamil, which we determined by the Tanimoto index (0.8) [83], it is likely that isoxsuprine binds to the verapamil binding site on the L-type voltage-dependent calcium channel. However, evidently it is necessary to model the multi-domain architecture of LVCC, using sophisticated methods and high-performance computing [84], in order to gain a better understanding of the effect of isoxsuprine and other small molecules on these channels.

Since diverse CVDs are associated with endothelial dysfunction related to NO/cGMP and H_2_S/K_ATP_ pathways impairment, it is important to search new leads for the development of alternative drugs, which are able to restore NO and H_2_S levels [53]. The results obtained in the present study provide evidence indicating that isoxsuprine induces a potent vasodilator effect that involves not only the activation of the NO/cGMP and H_2_S/K_ATP_ pathways, but also the blockade of calcium channels and α_1_-adrenoceptors. These mechanisms, which act synergistically, give this compound the possibility of producing significant beneficial clinical effects that clearly differ from that of conventional vasodilators. Our findings allow us to propose isoxsuprine as a very valuable drug that could be repurposed to treat a wide range of CVDs, such as hypertension, stroke, and heart failure.

## 4. Materials and Methods

### 4.1. Virtual Screening

#### 4.1.1. Human eNOS and CSE Enzyme Structures Preparation

The crystallographic structures of eNOS and CSE from *H. sapiens* were obtained from the Protein Data Bank, http://www.pdb.org [85,86,87]. In this work, the highest-resolution protein structures available of eNOS (PDB: 3NOS) [28] and CSE (PDB: 3COG) [29] were used. Cofactors for the active enzymes were included in these structures, which were prepared and corrected by the “Protein Preparation Wizard” (Schrödinger Release 2015-4: Schrödinger Suite 2015-4 Protein Preparation Wizard; Epik, Impact, Prime, Schrödinger, LLC, New York, NY, USA, 2015) [88]. Protein structures were optimized by adding missing atoms and amino acids, eliminating water molecules, accessory ions, and ligands, and choosing the best conformation for ambiguous side chains.

#### 4.1.2. Lead-Like Compounds Selection from Database and Preparation

Four libraries obtained from the ZINC database (http://zinc.docking.org/) [89,90,91] were used: (1) Natural Products (ZINC-Natural Products: 89,425 compounds), (2) Approved drugs (ZINC-DrugBank: 1731 compounds), (3) Commercial libraries (ZINC-Maybridge Commercial vendor library: 14,400 compounds) and (4) Diverse database of the U.S. National Cancer Institute (NCI Diverse: 1817 compounds). The four libraries were combined into a single comprehensive database with a total of 107,373 compounds, which was processed with MOE [24] to select compounds with a single SMILES code, thus unique structures were selected. The resulting database was subsequently processed by the LigPrep program (Schrödinger Release 2015-4: LigPrep, Schrödinger, LL, 2015) and a subset of lead-like molecules was obtained based on the following three properties: (1) molecular weight (MW), topological polar surface area (TPSA), and octanol-water partition coefficient (log P); (2) hydrogen bond acceptors and donors (HBA and HBD, respectively); and (3) molecular topology (number and size of rings, molecular flexibility, and number of rotatable bonds). The LigPrep program was also used to assign the protonation states of the compounds (pH = 7.0 ± 2.0) and to generate all tautomeric forms within this pH range. In the case of chiral molecules, chirality was retained, if specified, otherwise stereoisomers were generated. The 176,500 resulting structures were included in our lead-like library. 

#### 4.1.3. Virtual Docking

The HTVS Glide program [25,26,27] (Glide, version 6.2, Schrödinger, LLC, 2014) was used to find the 2000 structures that showed the highest affinity for each enzyme in the putative allosteric triterpene-binding site previously described for eNOS and CSE [18]. Then, the 2000 structures were redocked with four different programs: Glide XP [27], AutoDock [30], AutoDock Vina [31] and UCSF-Dock [32]. The nine compounds, which displayed the highest docking scores (“consensus hit compounds”) were selected for pharmacological evaluation (Figure 1).

### 4.2. Pharmacological Evaluation

#### 4.2.1. Reagents

Standards for the pharmacological and biochemical assays were purchased from Sigma-Aldrich (St. Louis, MO, USA). Isoxsuprine, carvedilol, propranolol, and pindolol were supplied as a racemic mixture. Stock solutions of water insoluble compounds were prepared in dimethyl sulfoxide (DMSO), where the highest concentration of this solvent in the incubation chamber was 0.2% (*v*/*v*). Other compounds and subsequent dilutions were prepared directly in distilled water. 

#### 4.2.2. Experimental Animals

All experiments were performed according to the NOM-062-ZOO-1999, “Technical specifications for the production, care, and use of laboratory animals”. Male Wistar (200–250 g) rats were provided by the Institute of Neurobiology of the National Autonomous University of Mexico (INB-UNAM), Campus Juriquilla. Protocol for animal use and handling was evaluated and approved by the Ethics Committee of the Faculty of Chemistry, Autonomous University of Querétaro (CBQ16/1116-7).

#### 4.2.3. Isolated Rat Aorta Assay and Participation of the NO/cGMP and the H_2_S/K_ATP_ Pathways in the Vasodilator Effect Elicited by the “Consensus Hits”

The isolated rat aorta assay was carried out according to the method previously reported [18,92,93]. Rats were sacrificed by decapitation using a guillotine (NOM-062-ZOO-1999, section 9.5.3.3). Then, thoracic aorta was removed and placed in a cold Krebs-Henseleit solution with the following composition (mM): 126.8 NaCl; 5.9 KCl; 1.2 KH_2_PO_4_; 1.2 MgSO_4_; 5.0 D-glucose; 30 NaHCO_3_; 2.5 CaCl_2_ (pH 7.4), bubbled with carbogen (95% O_2_ and 5% CO_2_). Adipose and connective tissues were removed from the aorta and thereafter, it was cut into 4–5 mm rings. Aortic rings were mounted in 5 mL incubation chambers with Krebs-Henseleit solution at 37 °C and constant bubbling with carbogen. Tissues were stabilized for 30 min under a tension of 1.5 g at 37 °C. During this period, the bathing medium was changed every 10 min. Once the basal tension was restored at 1.5 g, the aortic segments were contracted with KCl (100 mM) to sensitize the tissue. When the contraction with KCl was stable, the bath medium was replaced until the basal tension of 1.5 g was recovered. Subsequently, tissues were contracted with L-phenylephrine (Phe, 1 μM) and its contractile force was defined as 100%. Thereafter, the test compounds (0.001 to 1000 µg/mL) were cumulatively added to the chambers, 20 min after the addition of Phe. Sodium nitroprusside (SNP), sodium hydrosulfide (NaHS), and acetylcholine (ACh) were used as positive controls. The integrity of endothelium was periodically evaluated in a representative segment of aorta by determining the relaxation induced by ACh (1 μM; greater than 60%). Changes in aortic tonus caused by the test compounds were detected by Grass FT03 force transducers coupled to a Grass 7D Polygraph and were expressed as percentages of relaxation based on the contraction generated by adding Phe. Participation of the NO/cGMP and the H_2_S/K_ATP_ pathways in the vasodilator effect of the consensus hits was assessed by incubating the aortic rings for 20 min in the presence of 100 µM NG-nitro-L-arginine methyl ester (L-NAME, inhibitor of NOS) and 10 mM DL-propargylglycine (PAG, inhibitor of CSE) [18,92,93].

#### 4.2.4. Participation of Endothelium, the CO/cGMP and the PGI_2_/cAMP Pathways, Potassium Channels and β_2_ Adrenoceptors in the Vasodilator Effect of Isoxsuprine

In experiments with endothelium denuded aortic rings, endothelial cells were chemically removed with 0.2% deoxycholic acid. The absence of endothelium was confirmed by adding ACh (1 μM), which did not induce more than 5% of relaxation. In order to further investigate the mechanism of action of isoxsuprine, aortic rings were incubated for 20 min in the presence of the following compounds: (a) 15 μM chromium mesoporphyrin IX (CrMP, inhibitor of heme oxygenase, HO), (b) 10 μM indomethacin (Indo, inhibitor of cyclooxygenase, COX), (c) 10 μM glibenclamide (inhibitor of ATP-dependent potassium channels, K_ATP_) [18,92,93], and (d) 1 μM propranolol (a non-selective antagonist of β_1_ and β_2_ adrenoceptors).

#### 4.2.5. Participation of Blockade of α_1_-Adrenoceptors and Calcium Channels in the Vasodilator Effect of Isoxsuprine

To evaluate whether the blockade of α_1_-adrenoceptors contributes to isoxsuprine-induced vasodilation, concentration-response curves were obtained by noncumulative administrations of increasing concentrations of phenylephrine (from 1 × 10^−8^ to 1 × 10^−3^ M; 5 min contact), a selective α_1_-adrenoceptor agonist as the contracting compound, either alone or 5 min after the administration of isoxsuprine (1 × 10^−6^ M) or prazosin (1.2 × 10^−7^ M), a selective α1-adrenoceptor antagonist. The test compounds, either isoxsuprine or prazosin, were washed out of the tissue after each concentration of phenylephrine and then reapplied 5 min before the next concentration of the agonist. Changes in aortic tonus caused by phenylephrine were expressed as percentages of contraction based on the maximum contraction generated by adding this agonist [67].

In order to determine if isoxsuprine-induced vasodilation involved the blockade of L-type voltage-gated calcium channels, the rat aortic segments were stabilized in the Krebs-Henseleit solution, and afterwards calcium was removed, replacing the bathing medium with calcium-free solution containing EDTA (0.1 mM). Next, the solution was replaced with 60 mM KCl in calcium-free Krebs-Henseleit solution. Then cumulative concentration-response curves for CaCl_2_ (1 µM to 3 mM) were constructed in the absence (control) or presence of isoxsuprine (0.03 µg/mL) or verapamil (1 µM, positive control) [94].

#### 4.2.6. Measurement of H_2_S Levels in Rat Aorta Homogenates

Aortic segments were frozen in liquid nitrogen and homogenized in PBS pH 7.4 with a protease inhibitor (Sigmafast protease inhibitor cocktail tablets, EDTA free). Next, aorta homogenate (100 µL) and all components of the incubation mixture [pyridoxal-5’-phosphate (2 mM final concentration), L-cysteine (10 mM final concentration) and either isoxsuprine (0.026 ± 0.003 µg/ml final concentration) or isoxsuprine plus PAG (10 mM final concentration)] were poured to 2 mL vials fitted with septum stoppers and plastic center wells. Center wells were filled with 0.5 mL of 1% (*w*/*v*) zinc acetate pH 10 and a folded 2 cm × 2.5 cm rectangle of Whatman no. 1 filter paper for trapping evolved H_2_S. Each vial was flushed with N_2_ for 20 s and then sealed. This mixture was left to react for 60 min at 37 °C. The reaction was ended by adding 50% trichloroacetic acid and incubated for 60 min at 37 °C. Afterwards, 50 µL of 20 mM *N*,*N*-diethyl-*p*-phenylenediamine sulphate in 1.2 M HCl were added, followed by addition of 50 µL of 30 mM iron trichloride in 1.2 M HCl. After 20 min, absorbance was measured at 670 nm and H_2_S concentration was calculated against a calibration curve of standard NaHS solution (0–100 µM) [95].

#### 4.2.7. Statistical Analysis

Six evaluations were carried out for each concentration of the tested compounds. The results are expressed as the mean ± standard error of the mean (SEM). Experimental data were fitted to a sigmoidal equation, plotted and analyzed to calculate EC_50_ and Emax values (GraphPad Prism 7.02, San Diego, CA, USA). These results were subjected to one-way ANOVA analysis, followed by Dunnett’s post hoc test, using the statistical program GraphPad Prism 7.02. Values of * *p* < 0.01, ** *p* < 0.001, *** *p* < 0.0001 were considered to be significant. In Figure 7, statistical analysis was made using a one-way ANOVA, followed by a Tukey’s test. In Figure 8B, statistical analysis was made using t-test with Welch´s correction.

### 4.3. Virtual Pharmacophoric Elements Identification and General Protein-Ligand Interaction Model

The software Pharmer (2015, Pittsburg, PA, USA) [96] was used to identify the key pharmacophoric elements of the consensus hits on their protein-ligand complexes: hydrogen bond donors and acceptors, aromatic systems, hydrophobic groups, and ionic groups in the three-dimensional conformations of the ligands. These characteristics were clustered according to their nature, position, and size using a partition around medoids statistical method [97], implemented in the cluster package [98], available in the statistical software R (3.5.2, Vienna, Austria) [99]. 

### 4.4. Molecular Dynamics (MD) of Isoxuprine-CSE Interaction

Once the CSE-isoxuprine structure was obtained, this complex was subjected to molecular dynamics simulation with GROMACS 5.1.4 [100], using the AMBER99SB force field [101] and adjusting the parameters necessary for the ligand with ACPYPE [102]. The protonation state was defined at physiological pH, so that the ligand had a positive charge. Electrostatic and Lennard-Jones interactions had a cut-off of 1 nm. Simulation was carried out in periodic conditions by using rectangular boxes of maximum length of the system plus 1 nm. The complex was subjected to a minimization of energy followed by a period of equilibrium of free and restricted position, to finally carry out an isothermal-isobaric (300 K, 1 atm) computer simulation of 10 ns, employing a temperature coupling and velocity rescaling with a stochastic term [103] and a Parrinello-Rahman barostat [104]. Binding free energy was calculated by means of the LIE method [105] as a notion of affinity of the ligand for the enzyme.

## 5. Conclusions

In conclusion, in this work we present for the first time evidences indicating that the vasodilation induced by isoxsuprine comprises different mechanisms that include activation of the NO/cGMP and the H_2_S/K_ATP_ pathways and blockade of α_1_-adrenoceptors and L-type voltage gated calcium channels. All these mechanisms act in a synergistic manner to produce a potent vasodilator effect. This study presents valuable elements for the repositioning of isoxsuprine as a promising molecule that could be used in the therapeutics of various cardiovascular pathologies. Finally, our results provide support for the usefulness of the triterpene-binding putative allosteric sites on eNOS and CSE as valuable pharmacological targets in the search of hit compounds for the development of drugs useful to treat cardiovascular diseases.

## Figures and Tables

**Table 1:** EC_50_ values of the consensus hits and the positive controls.

Compound	EC_50_ (µM) ± SEM	E_max_ (%) ± SEM
**Controls**		
SNP	0.0099 ± 0.001	100.7 ± 0.747
ACh	50.30 ± 5.126	68.46 ± 1.122
NaHS	191.0 ± 9.841	92.17 ± 2.721
**Highly Potent Compounds**		
Isoxsuprine	0.046 ± 0.004	106.1 ± 0.343
Carvedilol	0.069 ± 0.003	106.6 ± 0.115
Nebivolol	2.014 ± 0.215	106.7 ± 0.543
**Lowly Potent Compounds**		
Propranolol	18.120 ± 1.419	103.6 ± 1.663
Pindolol	39.490 ± 2.603	106 ± 0.463
Sitagliptin	252.30 ± 8.058	117.6 ± 0.497
Fenoterol	608.60 ± 43.225	120.3 ± 1.548
Epicatechin	626.40 ± 67.372	101.5 ± 2.400
Midodrine	4698 ± 324.691	219.5 ± 8.219

Data are means ± SE, relaxation is expressed as a percentage of the precontraction induced by 1 μM phenylephrine; *n* = 6.

**Table 2:** EC_50_ values of the “consensus hits” and participation of the NO/cGMP and the H_2_S/K_ATP_ signaling pathways in their vasodilator effect.

Compound	Control	L-NAME	PAG
	EC_50_ (µM) ± SEM	EC_50_ (µM) ± SEM	EC_50_ (µM) ± SEM
**Most Potent Vasodilators**			
Isoxsuprine	0.0461 ± 0.004	0.3846 ± 0.040 ***	0.3255 ± 0.026 ***
Carvedilol	0.0695 ± 0.003	0.3643 ± 0.011 ***	0.1500 ± 0.019 **
Nebivolol	2.0135 ± 0.215	11.290 ± 1.501 ***	2.5280 ± 0.229
**Less Potent Vasodilators**			
Propranolol	18.120 ± 1.419	40.430 ± 5.696 **	27.900 ± 3.651
Pindolol	39.490 ± 2.603	166.1 ± 3.610 ***	89.47 ± 7.940 ***
Sitagliptin	252.30 ± 8.058	864.3 ± 8.692 ***	436.2 ± 12.63 ***
Fenoterol	608.6 ± 43.225	882.1 ± 27.513 ***	1038 ± 22.241 ***
Epicatechin	626.4 ± 67.372	1789 ± 389.469 *	3087 ± 552.718 **
Midodrine	4698 ± 324.691	8618 ± 2060.477 *	10076 ± 1955.342 *

Data are means ± SEM (*n* = 6). Statistical analysis was made using a one-way ANOVA, followed by Dunnett’s post hoc test. Values of * *p* < 0.01, ** *p* < 0.001, *** *p* < 0.0001 were considered as significant.

## Supplementary Materials

The Supplementary Materials are available online. Figure S1: CSE-isoxsuprine and CSE-fenoterol binding free energies results (LIE Method) obtained from Molecular Dynamics Simulation with GROMACS, using AMBER force field and adjusting the parameters required for the ligand with ACPYPE. Table S1: Docking scores by program and percentile consensus of the “consensus hit compounds”.
