# Supplementary material for: Vasodilation Elicited by Isoxsuprine, Identified by High-Throughput Virtual Screening of Compound Libraries, Involves Activation of the NO/cGMP and H2S/KATP Pathways and Blockade of α1-Adrenoceptors and Calcium Channels
Source: Molecules. 2019 Mar 11;24(5):987. doi: 10.3390/molecules24050987 (PMC6429095; doi:10.3390/molecules24050987)
Supplement: Supplementary file 1 [file molecules-24-00987-s001.pdf]

Table 1. Docking scores by program and percentile consensus of the "consensus hit compounds".

Consensus hit compounds	AD	Glide	Vina	D1	D2	C	AD	Glide	Vina	D1	D2	C
	**eNOS (ΔG°, kcal/mol)**						**CSE (ΔG°, kcal/mol)**					
**Nebivolol**	-12.55	-8.16	-8.4	-231	122.08	0.02	-11.16	-6.11	-7.2	-196	-36.68	0.02
**Carvedilol**	-11.38	-7.785	-6.7	-279	-2.683	0.05	--11.62	-6.294	-6.9	-168	-13.164	0.09
**Fenoterol**	-10.38	-7.798	-7.9	-255	-12.507	0.05	-8.31	-6.62	-6.2	-160	-21.05	0.023
**Propranolol**	-9.87	-6.625	-7.4	-220	39.78	0.17	-9.29	-4.675	-6.7	-135	-22.099	0.30
**Midodrine**	-7.68	-7.682	-7.3	-214	97.38	0.20	-6.69	-4.865	-5.5	-149	-21.88	0.31
**Pindolol**	-9.66	-6.54	-7.1	-199	27.858	0.25	-7.72	-4.872	-6.1	-88	9.469	0.34
**Isoxsuprine**	-11.38	-6.035	-7.6	-176	176.391	0.25	-8.91	-5.873	-6.1	-120	-13.856	0.44
**Sitagliptin**	-10.01	-6.023	-7.0	-241	22.632	0.26	-8.09	-5.156	-7.0	-175	-15.915	0.27
**Epicatechin**	-9.82	-5.834	-6.6	-235	-3.102	0.30	-7.15	-4.65	-7.1	-151	-19.092	0.26

AD: AutoDock 4.2, Glide: Glide XP, D1: Dock1 (UCSF-Dock), D2: Dock2 (UCSF-Dock), C: Consensus

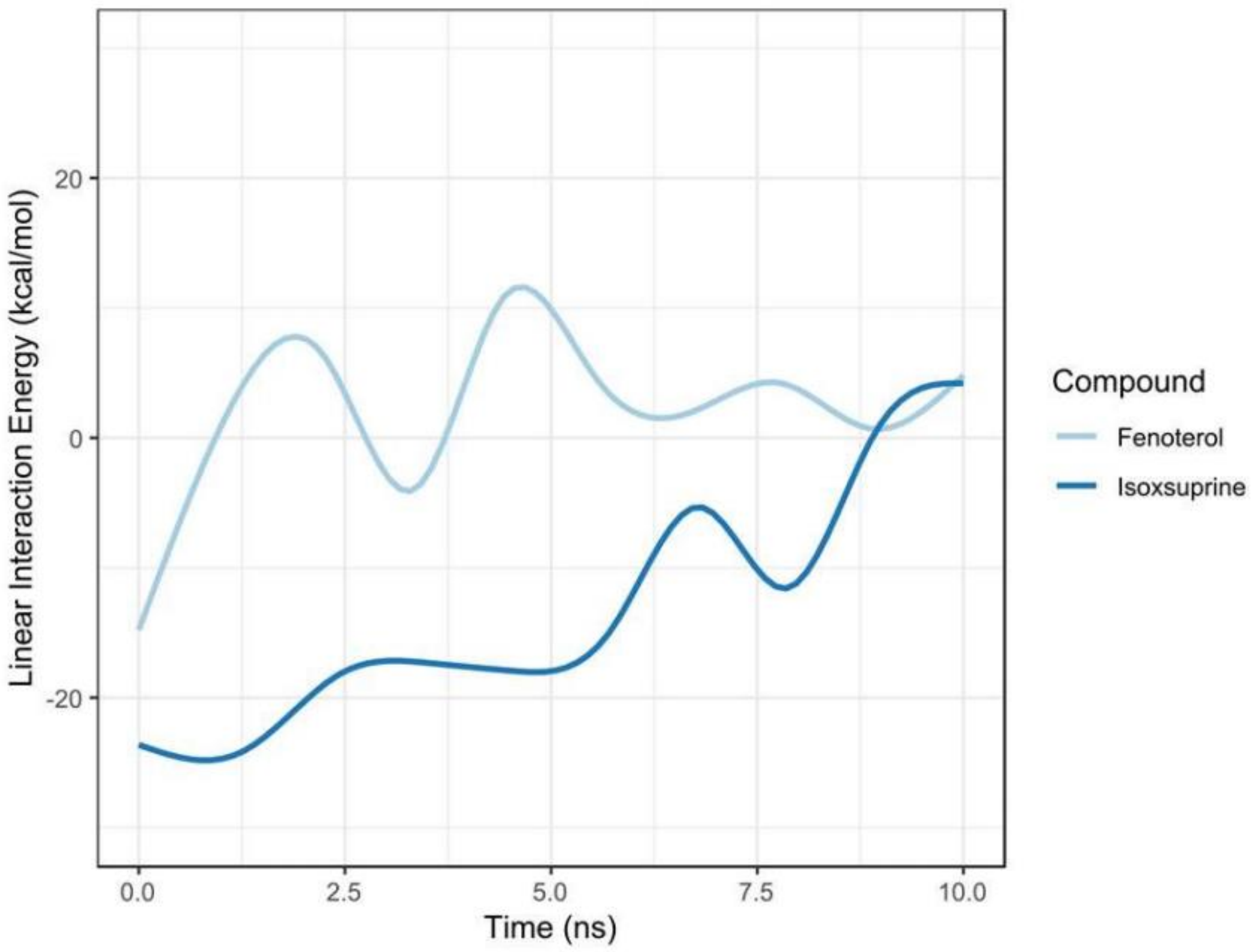

Figure 1. CSE-isoxsuprine and CSE-fenoterol binding free energies results (LIE Method) obtained from Molecular Dynamics Simulation with GROMACS, using AMBER force field and adjusting the parameters required for the ligand with ACPYPE.
